# *Bartonella* spp. DNA Associated with Biting Flies from California

**DOI:** 10.3201/eid1007.030896

**Published:** 2004-07

**Authors:** Crystal Y. Chung, Rickie W. Kasten, Sandra M. Paff, Brian A. Van Horn, Muriel Vayssier-Taussat, Henri-Jean Boulouis, Bruno B. Chomel

**Affiliations:** * University of California, Davis, California, USA;; † Unité Mixte de Recherche, Ecole Nationale Veterinaire d'Alfort, Maisons-Alfort, France

**Keywords:** *Bartonella*, *Chrysops*, *Haematobia*, *Stomoxys*, *Tabanus*, Ruminants

## Abstract

*Bartonella* DNA was investigated in 104 horn flies (*Haematobia* spp.), 60 stable flies (*Stomoxys* spp.), 11 deer flies (*Chrysops* spp.), and 11 horse flies (*Tabanus* spp.) collected on cattle in California. Partial sequencing indicated *B. bovis* DNA in the horn fly pool and *B. henselae* type M DNA in one stable fly.

*Bartonella* spp. are vector-borne bacteria associated with numerous emerging infections in humans and animals (1). Four *Bartonella* species have been isolated from wild and domestic ruminants. *B. schoenbuchensis* and *B. capreoli* were recovered from wild roe deer (*Capreolus capreolus*) (2,3) in Europe, whereas *B. bovis* (formerly *B. weissii*) was recovered from domestic cattle in the United States and Europe (3–5). Strains similar to *B. bovis* and *B. capreoli* were also isolated from mule deer (*Odocoileus hemionus*) and elk (*Cervus elaphus*) from California (3,4). Recently, *B. chomelii* was recovered from bacteremic cows in France (6). A high prevalence of infection with various *Bartonella* species has been reported in domestic and wild ruminants in North America and Europe (2–4). Of the herds investigated in California, 95% of beef cattle and 17% of dairy cattle were bacteremic for *B. bovis* and 90% of the mule deer were bacteremic for *Bartonella* spp (4). The main vector of these ruminant-infecting *Bartonella* spp. has not been identified.

The role of ticks as potential vectors for *Bartonella* in cattle was investigated (7,8). In Europe, >70% of 121 *Ixodes ricinus* ticks collected from roe deer had 16S rRNA gene sequences for *Bartonella* or other closely related species (7). In California, *Bartonella* DNA was detected in approximately 19% of 151 questing adult *I. pacificus* ticks (8), but the direct role of ticks in *Bartonella* transmission among ruminants has never been established. In a search for an efficient *Bartonella* vector, which could explain such high prevalence of infection in wild and domestic ruminants, we tested biting flies for *Bartonella* spp. DNA to establish the potential role of biting flies as vectors of *Bartonella* in cattle.

## The Study

Flies were collected by hand, with a bug net, at various locations on the University of California campus, mainly the dairy barn, beef barn, and feedlot, from early July to mid-August 2003. Flies were identified on the basis of morphologic characteristics visually or under binocular lenses for the smaller flies by an experienced entomologist. Of the 370 biting flies collected, 104 (62%) of the horn flies (*Haematobia* spp.), 60 (33%) of the stable flies (*Stomoxys* spp.), 11 (92%) of the deer flies (*Chrysop*s spp.), and 10 (91%) of the horse flies (*Tabanus* spp.) were tested for *Bartonella* DNA. The stable flies were collected from the dairy and the feedlot barns. The horn flies, deer flies, and horse flies were collected from the beef barn.

Before DNA extraction, the flies were placed in a sterile 1.5-mL microtube, washed with 70% ethanol, and rinsed with sterile water. Because of size differences among the flies, 2–3 horn flies were grouped together in a single microtube, while each stable fly was placed in an individual vial. The abdomen of deer flies and horse flies was first removed and then placed in individual vials. DNA extraction was performed by using the DNeasy Tissue Kit (Qiagen, Valencia, CA) according to the manufacturer's instructions, with some minor adjustments. The amount of reagents for the deer and horse flies were doubled, and the flies were incubated in a waterbath overnight at 55°C.

*Bartonella* DNA was detected by polymerase chain reaction (PCR) using primers for the citrate synthase (*gltA*) gene, as previously published (9). Undiluted DNA extracted from the flies was used as the DNA template. As a positive control, a low concentration of *B. henselae* was added to a separate set of the same DNA template. A negative control was made by using sterile water instead of the DNA template. Using gel electrophoresis, we analyzed PCR products for the appearance of an ≈380-bp fragment. Any evidence of a 380-bp fragment was further analyzed by restriction fragment length polymorphism (RFLP) procedures, by using *Taq*I (Promega Corp., Madison, WI), *Hha*I, *Aci*I, and *Mse*I endonucleases (New England Biolabs, Beverly, MA), and DNA sequence analysis (Davis Sequencing, Davis, CA).

**Figure 1:**
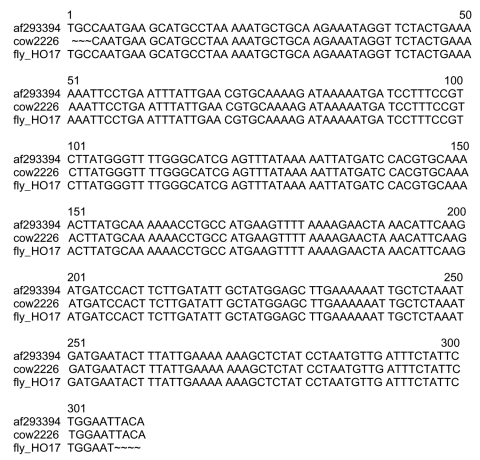
Alignment of BhCS.781p/BhCS.1137n *gltA* gene amplicons for 306 bp of *Bartonella bovis* (GenBank accession no. af293394), a *B. bovis* isolate (cow 2226) from a Californian cow and the horn fly pool (fly-HO17).

**Figure 2:**
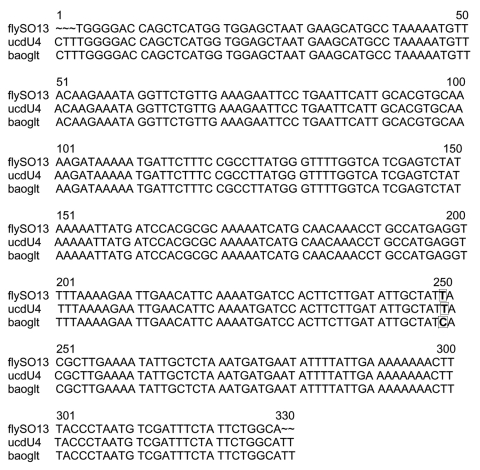
Alignment of BhCS.781p/BhCS.1137n *gltA* gene amplicons for 328 bp of *Bartonella henselae* type H (GenBank accession no. baoglt), *B. henselae* type M (isolate ucd-U4) from a California cat and the stable fly DNA extract (fly-SO13). The highlighted region indicates base pair difference.

Four of the 60 stable flies and one pool (2 flies) of the 45 horn fly pools showed a 380-bp fragment. PCR/RFLP analysis confirmed *Bartonella* DNA in one of the four stable flies and in the horn fly pool. However, for the three other stable flies, the PCR/RFLP profiles did not match any known *Bartonella* digestion profile. The sequence obtained from the horn fly pool (*Haematobia* spp.) collected in the beef cattle barn was identical to that for *B. bovis* (Figure 1). The sequence obtained from a stable fly (*Stomoxys* spp.) collected in the dairy cattle barn was identical to that for *B. henselae* type M (Marseille) (Figure 2). The highlighted area indicates the divergence between *B. henselae* type H (Houston I) and *B. henselae* type M, as previously described (10).

## Conclusions

This identification of *Bartonella* DNA is the first associated with horn and stable flies and the first identification of *B. henselae* from a biting fly. It is also the first report of identification of *Bartonella* DNA from flies from North America. This finding demonstrates, as for ticks, that *Bartonella* DNA is present in various biting insects. We found a very low percentage of *Bartonella* DNA–positive flies, in contrast to the very high prevalence (57 [88%] of 65 observed in *Hippoboscidae* adult flies (*Lipoptena cervi* and *Hippobosca equina*) collected from domestic cattle and wild roe deer in France (H.J. Boulouis, pers. comm.). This low prevalence may be related to the fact that different fly species were tested but more likely could be associated with a low level of *Bartonella* bacteremia in our herds. In a previous study, only 17% of cows in a dairy herd were bacteremic (4), and prevalence was even lower in another dairy herd from Tulare, in the central valley of California (B.B. Chomel et al., unpub. data). A follow-up for this study would be to collect blood from herds at the University of California, Davis, and establish the status of *Bartonella* bacteremia. Future research should include collecting flies in different locations and herds in which high levels of bacteremia were previously detected. Inhibitory factors were unlikely to be associated with such a low prevalence because spiked controls were systematically detected.

Identification of *B. henselae* DNA in a stable fly indicates the wide range of blood-sucking arthropods that can harbor this human pathogen. The partial *gltA* sequence was identical to that for *B. henselae* type Marseille, the most common type found in cats and humans in California (11). Fleas have been shown to be an efficient vector of *B. henselae* (12–14). More recently, *B. henselae* DNA was identified in adult questing *I. pacificus* ticks from California and from *I. ricinus* ticks collected on humans in Italy (8,15). The role of ticks as potential vectors of *B. henselae* in humans has also been suggested (16–18). Since *Bartonella* are likely to be present in biting flies, investigating the potential of biting flies as either mechanical or biologic vectors of *Bartonella* in cattle and possibly humans should be pursued.
